# A Novel Sleep Respiratory Rate Detection Method for Obstructive Sleep Apnea Based on Characteristic Moment Waveform

**DOI:** 10.1155/2018/1902176

**Published:** 2018-01-10

**Authors:** Yu Fang, Zhongwei Jiang, Haibin Wang

**Affiliations:** ^1^Graduate School of Science and Engineering, Yamaguchi University, Yamaguchi, Japan; ^2^School of Electrical Engineering and Electronic Information, Xihua University, Chengdu, China

## Abstract

Obstructive sleep apnea (OSA) affecting human's health is a kind of major breathing-related sleep disorders and sometimes leads to nocturnal death. Respiratory rate (RR) of a sleep breathing sound signal is an important human vital sign for OSA monitoring during whole-night sleeping. A novel sleep respiratory rate detection with high computational speed based on characteristic moment waveform (CMW) method is proposed in this paper. A portable and wearable sound device is used to acquire the breathing sound signal. And the amplitude contrast decreasing has been done first. Then, the CMW is extracted with suitable time scale parameters, and the sleep RR value is calculated by the extreme points of CMW. Experiments of one OSA case and five healthy cases are tested to validate the efficiency of the proposed sleep RR detection method. According to manual counting, sleep RR can be detected accurately by the proposed method. In addition, the apnea sections can be detected by the sleep RR values with a given threshold, and the time duration of the segmentation of the breath can be calculated for detailed evaluation of the state of OSA. The proposed method is meaningful for continued research on the sleep breathing sound signal.

## 1. Introduction

Humans spend almost 30% of the time in sleeping, and the sleep quality is very important for human's health. Breathing-related sleep disorders are characterized by abnormalities of the respiratory pattern or the quantity of ventilation during sleep [1]. It is considered a chronic illness which needs long-term treatment and management. Obstructive sleep apnea (OSA) is a kind of major breathing-related sleep disorders, and it is described by full or partial occlusion of the upper airway during sleep which can produce repeated oxyhemoglobin desaturations and sleep fragmentation [2]. OSA which is considered a main risk factor for cardiovascular disease affects human's health and sometimes leads to nocturnal death [3, 4].

OSA is commonly defined as a minimum of 10 s interval pause of breath. The Apnea-Hypopnea Index (AHI) is described by the number of apnea and hypopnea events per hour to assess OSA severity. AHI of 5–15 indicates mild OSA; 15–30, moderate; and over 30, severe [5].

Sleep respiratory rate (RR) is an important indicator for serious illness [6], especially for OSA monitoring. RR of healthy adults in a relax state is about 12–20 times per minute. However, the RR will be abnormal for the OSA case while the sleep breathing becomes slowed or stopped by the apnea [7]. Hence, sleep RR is an early and vital indicator for OSA patients.

Polysomnography (PSG) is often used to detect OSA in clinic which acquires a series of monitoring indices including RR. But PSG with many sensors is not only expensive but also complicated for common patients [8]. Moreover, it is uncomfortable for the testers during their sleep, so the results of PSG will be influenced by the low-quantity sleep of the testers. With the development of a smart wearable device, several researchers have interests in RR detection by acoustic signals.

The acoustic signals mainly come from two aspects, breathing sound signals of the nose and the mouth [9] and tracheal signals from the throat [10] and the suprasternal notch [6, 11]. For RR detection via a tracheal signal, Hilbert transform was applied to extract the sound envelope and wavelet was applied for frequency content decomposition with a success rate of 96% for healthy volunteers and 85% for patients suffering from chronic pulmonary diseases [10]. A respiratory phase segmentation method based on a genetic algorithm was applied to monitor the RR which was enhanced by exploiting the signal redundancy [11]. The short-time Fourier transform, Shannon entropy, and autocorrelation were calculated to detect the RR value [6]. It is found that the previous RR detection methods are mainly based on the tracheal signal, and the acquisition of the tracheal signal is not convenient as a sleep breathing sound signal. And the selection of a threshold value which plays an important role in envelope extraction will change accompanied by the speed of breathing for different individuals. So the adaption of the threshold values, that is, the time scale parameters, will affect the accuracy of sleep RR detection and should be solved for further research. The OSA monitoring should be completed all night, and the results of sleep RR detection need to be transferred to an analysis system correctly and timely. In the previous research, the RR estimation via finding the largest spectral peaks of autoregressive power spectral analysis has been proposed [9]. And the successful rates for the patients' RR detection by the breathing sound from the mouth and nose were 85% and 84%, respectively [10]. They are not effective for the OSA case with apnea and not satisfied with the practical demand. In this paper, a RR detection method via a sleep breathing sound signal based on characteristic moment waveform is proposed.

This paper is divided into 6 sections.  Section 2 introduces sleep breathing sound signal acquisition.  Section 3 describes details of the characteristic moment waveform extraction method.  Section 4 gives the introduction of the sleep RR detection method. The results and analysis are disclosed in Section 5, and conclusions will be drawn in Section 6.

## 2. Sleep Breathing Sound Signal Acquisition and Preprocessing

### 2.1. Acquisition System of the Sleep Breathing Sound Signal

**Figure 1:**
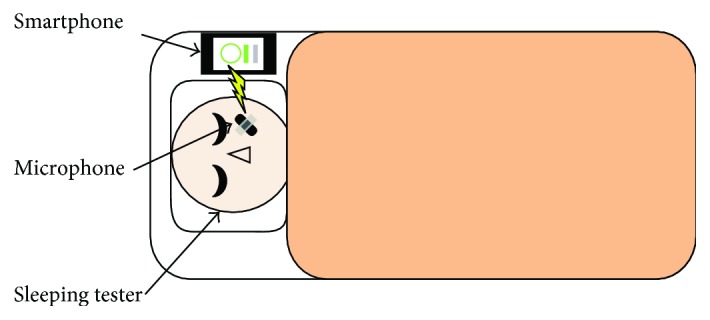
Sleep respiratory signal acquisition system.

The sleep breathing sound signal is collected by a portable and wearable acquisition device for high sleep quality, including a smart phone with an android system and a wireless microphone. The purpose of our research is to develop a cheap and easy-to-use sleeping monitoring system for home use, so that the commercial wireless headset (such as PTM 165) will be one better choice for our research. Compared with the acquisition positions inferred, the microphone is fixed near the nose by a kind of makeup tape to acquire a stable breath signal during whole-night sleeping. The environment of data acquisition is shown in Figure 1. The original sample frequency is 44.1 kHz.

### 2.2. Preprocessing for Amplitude Contrast Diminution

In fact, the intensity of the sleep breathing sound signal will change greatly and impact the efficiency of the proposed sleep RR detection method. The weak breathing sound will be covered by the heavy breathing and the surrounding noise. Therefore, the amplitude contrast of different breathing cycles should be decreased first. The enhanced preprocessing method is first introduced in detail as follows. The entropy of the original signal *H*(*t*) is defined as

(1) $$\begin{array}{c} H\left(t\right)=E\left[y\left(t\right)\right]=-\xi y\left(t\right)\cdot ln\left(y\left(t\right)\right),\;\left\{\begin{array}{l} \xi =-1\left(y\left(t\right)>0\right)\\ \xi =0\left(y\left(t\right)=0\right)\\ \xi =1\left(y\left(t\right)<0\right). \end{array}\right. \end{array}$$

Then, decrease the volume and intensity difference by cutting off the strong intensity part; the output signal is

(2) $$\begin{array}{c} H_{cut}\left(t\right)=a\cdot H\left(t\right)\pm b\cdot av\left(\left|H\left(t\right)\right|>av\right),\\ H_{cut}\left(t\right)=c\cdot H\left(t\right)\left(\left|H\left(t\right)\right|<av\right), \end{array}$$

where *av* is the mean value of the *H*(*t*), *a* and *b* are weakening factors, and *c* is the enhancement factor.

According to the experimental results by trial and error, *a* is selected as 0.4, *b* is 0.6 when *H*(*t*) is positive and −0.6 when *H*(*t*) is negative, and *c* is set as 1.5 to enhance the amplitude of a weak breathing cycle.

The final preprocessed signal is given by

(3) $$\begin{array}{c} y_{enhance}\left(t\right)=H_{cut}\left(t\right)\cdot \left(1-l\right)+l\cdot H_{cut}{\left(t\right)}^{N}, \end{array}$$

where *l* experimentally set as 0.85 is the limiting amplitude factor and *N* is set as 20 by experience.

**Figure 2:**
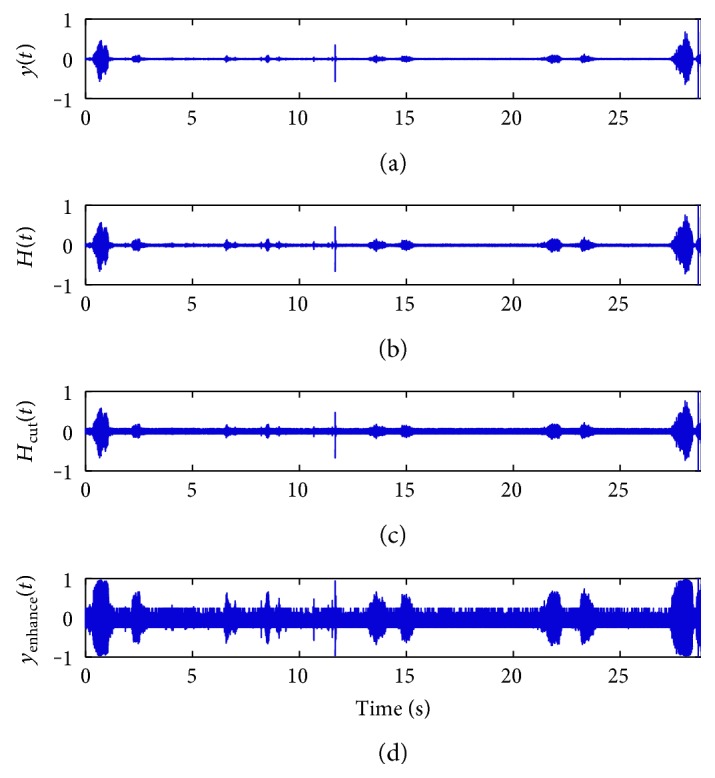
Sleep breathing sound signal waveforms, (a) original signal waveform, and (b–d) the procedure of the preprocessing.

A section of the sleeping breathing sound signal with large intensity variation is shown in Figure 2(a). Compared with the cycles in the both ends, the amplitude of three breathing cycles in the middle is too small to be detected. And after a series of processing shown in Figures 2(b) and 2(c), it is clearly found that the amplitude contrast of each breathing cycle has been shrunk shown in Figure 2(d), and it will improve the accuracy of the sleep RR detection.

## 3. Characteristic Moment Waveform Extraction of the Breathing Sound

A sleep breathing sound signal is generated by the movement of air through the respiratory system, the nose, and the mouth. It is always affected by a tester's healthy condition, mental state, sleeping environment, and so on. It is considered a quasiperiodic signal, and the sleep RR index can be computed by counting the number of the breathing period per minute in clinic.

### 3.1. Characteristic Moment Waveform (CMW)

Waveform extraction is always applied at the beginning of the signal processing in a time domain. The waveform should keep the useful information of the sleep breathing sound signal as much as possible and make the impaction of noise as less as possible. Commonly, Hilbert transform and Shannon entropy are used for waveform extraction [10, 12, 13]. According to the features of the biomedical signals, one single freedom model [13], a homomorphic filter [14], and other means are also applied for extracting the waveform. In this paper, the time characteristic waveform (TCW) is extracted first with multiscale adjustment. And then, the characteristic moment waveform (CMW) is proposed for sleep RR detection based on TCW.

The precondition is assuming the noise part of the sleep breathing sound signal as the signal with zero mean and unit variance. Suppose the sleep breathing sound signal is *r*(*t*), the random noise signal is *n*(*t*), and the real output signal is *y*(*t*) *= r*(*t*) *+ n*(*t*). TCW of the sleep breathing sound signal, marked as *c*(*t, δ*), defined as the variance of the output *y*(*t*) can be gotten by

(4) $$\begin{array}{c} c\left(t,\delta \right)=\sigma^{2}\left(y\right)=\int_{t-\delta }^{t+\delta }{\left(y\left(\tau \right)-\bar{y}\left(t\right)\right)}^{2}d\;\tau =\int_{t-\delta }^{t+\delta }y{\left(\tau \right)}^{2}d\;\tau -2\delta \bar{y}{\left(t\right)}^{2},\\ \bar{y}\left(t\right)=\frac{1}{2\delta }\int_{t-\delta }^{t+\delta }y\left(\tau \right)d\;\tau . \end{array}$$

Then, the CMW is calculated by the thought of image shape identification in image processing with another time scale *l*, which is represented by *I*(*t*, *δ*, *l*). It is calculated as follows:

(5) $$\begin{array}{c} I\left(t,\delta ,l\right)=\int_{t-l}^{t+l}{\left(\tau -t\right)}^{2}c\left(\tau ,\delta \right)d\tau . \end{array}$$

And the normalization presentation is presented as

(6) $$\begin{array}{c} n\left(t,\delta ,l\right)=\frac{\int_{t-l}^{t+l}{\left(\tau -t\right)}^{2}c\left(\tau ,\delta \right)d\tau }{\int_{t-l}^{t+l}c\left(\tau ,\delta \right)d\tau }, \end{array}$$

where *δ* and *l* are neighborhood of time *t*, which is called the width time scale.

It is easy to find that the calculated amount will increase with a larger time scale *δ* and *l*. The integral waveforms are applied to compute the TCW and CMW. The calculations of TCW and CMW are independent of the time scale parameters and fast with a very simple algorithm, just using additions and multiplications [15].

### 3.2. Scale Choice for TCW and CMW

**Figure 3:**
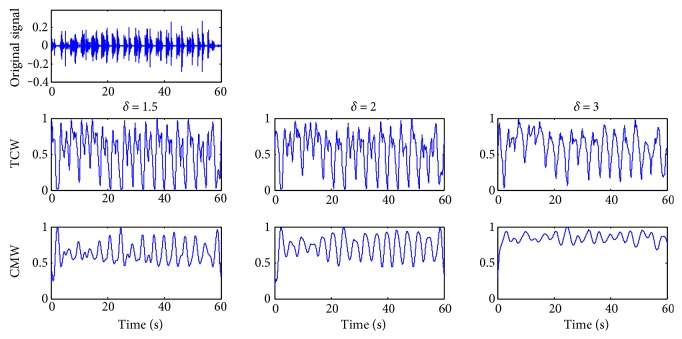
Time characteristic waveforms (TCW) and moment waveforms (CMW) of the breathing sound signal in the normal case while *l =* 0.1 and *δ* = 1.5, 2.0, and 3.0, respectively, from left to right.

A breathing cycle is constructed by four phases: inhalation, inspiratory pause, exhalation, and period of rest; the RR value is defined by the time duration during two consecutive inspirations [16]. According to our experimental statistic, a normal sleep breathing cycle is about 3 to 5 seconds and the time inspiration/expiration phase duration has a range of (0.3, 1) seconds. So the scale *δ* is usually set to (1.5, 3), about half of the sleep breathing cycle. The accuracy of CMW is not required in high level for sleep RR detection, and the time scale *l* is set as 0.1, about 1/10 of the phase duration. And the affection of the scale *δ* is shown directly in Figure 3.

**Figure 4:**
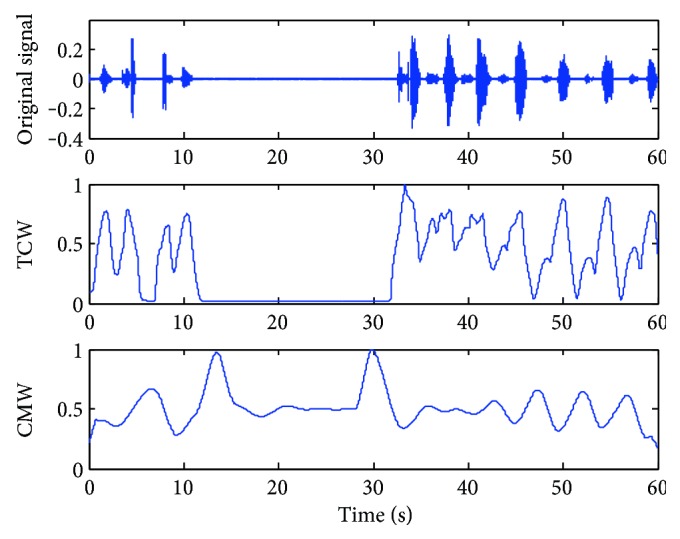
TCW and CMW of the breathing sound signal in the apnea case while *δ* = 2.5 and *l =* 0.1.

The TCW and CMW of a stable sleep breathing sound signal are shown in Figure 3 while *δ* is set as 1.5, 2, and 3, respectively. For this case, a sleeping breathing cycle lasts about 4 seconds and *δ* is set to 2.0 as the most suitable value based on the rules of the scale selection. While *δ* = 1.5, the waveforms of TCW and CMW are not smooth for the next segmentation. While *δ* = 3, the necessary details of the waveforms are ignored which weakens the periodicity. For the abnormal breathing case shown in Figure 4, *δ* is set to 2.5 as the breathing cycle lasting about 5 seconds.

In addition, according to the extracted waveforms, the most useful information of the original sleep breathing sound signal can be kept from the TCW waveform. And CMW with clear periodicity is convenient for finding the sleep RR index.

## 4. Respiratory Rate Detection Method

After choosing the suitable time scales, TCW and CMW are extracted according to (4), (5), and (6) and the sleep RR index can be detected using the following steps [15].

**Figure 5:**
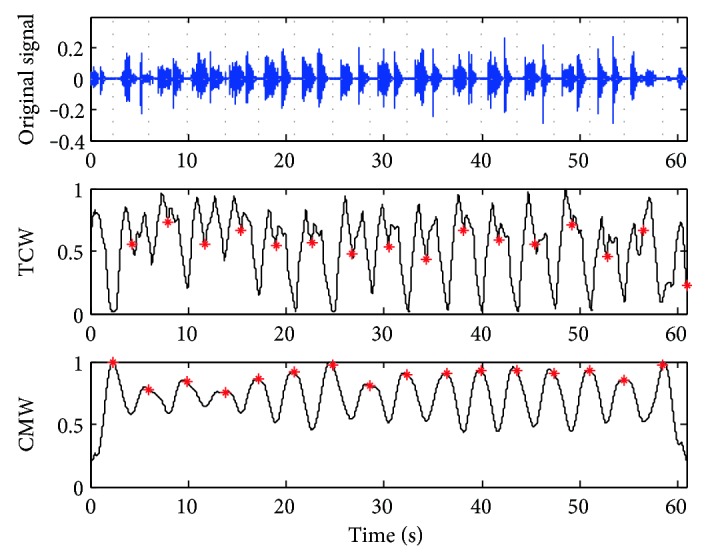
Results of the breath cycle segmentation of the case in Figure 3.

**Figure 6:**
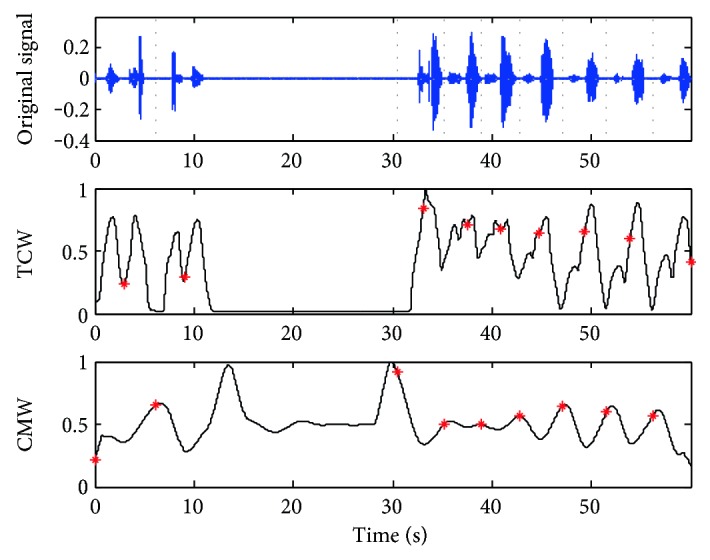
Results of the breath cycle segmentation of the case in Figure 4.

Step 1: Calculate the maximum point sequence of CMW.
Step 2: Find the local maximum point sequence by computing the maximum value of the point sequence gotten from Step 1.
Step 3: Calculate the local minimum point sequence of TCW shown in the middle plants of Figures 5 and 6.
Step 4: Adjust the cycle segment points by a computation window with the central point as the local minimum point sequence of TCW and the segment points shown in the bottom of Figures 5 and 6.
Step 5: Count the number of the cycle segment point per minute as the RR value.

Take the cases shown in Figures 3 and 4 for example; the breathing cycles are segmented correctly based on the TCW and CMW displayed by the gray dot line in Figures 5 and 6. Even there is some noise coming from the movements of the mouth, the segment results have not been affected. 16 breathing cycles in Figure 5 and nine breathing cycles in Figure 6 are extracted correctly. The proposed method shows outstanding stability and accuracy in sleep RR value detection.

## 5. Experiment

### 5.1. The Information of Experimental Data

Five young students (21 ± 1 years old) and a 59-year-old man who was diagnosed with OSA in the clinical setting are selected as testers.

Utilizing the acquisition system of the sleeping breathing sound signal, we recorded about 374-minute-length data and counted the breathing cycles manually with the guidance of the prodoctor for the reference. The information of the experimental data is listed in Table 1, and the OSA case is number 6.

### 5.2. The Efficiency of the Preprocessing

Through a series of processing introduced in Section 2.2, the intensity difference between strong and weak respiratory signals becomes small and its efficiency is validated.

The results of breathing cycle segmentation before and after applying the enhanced preprocessing method are summarized in Table 2. Without preprocessing, the scale parameters (*δ*, *l*) are selected as (2, 0.1), (2, 0.1), (2.5, 0.1), (2.5, 0.1), (3, 0.1), and (3, 0.1) for test cases orderly. While applying the enhanced preprocessing method, the scale parameters (*δ*, *l*) are set as (2.5, 0.1) for all cases.

From Table 2, it seems that the method without preprocessing can detect the breathing cycle with a success rate of at least 93.06%. And the total successful rate is improved to 98.40% with the same predicted time scale parameters for different cases when applying the enhanced preprocessing method. Especially, the successful rate of the OSA case that improved to 97.44% can satisfy the experimental requirement of the sleep RR detection. Therefore, the use of the enhanced preprocessing method shows more adaptability and veracity in this experiment.

### 5.3. The Sleep RR Detection for OSA Analysis

**Figure 7:**
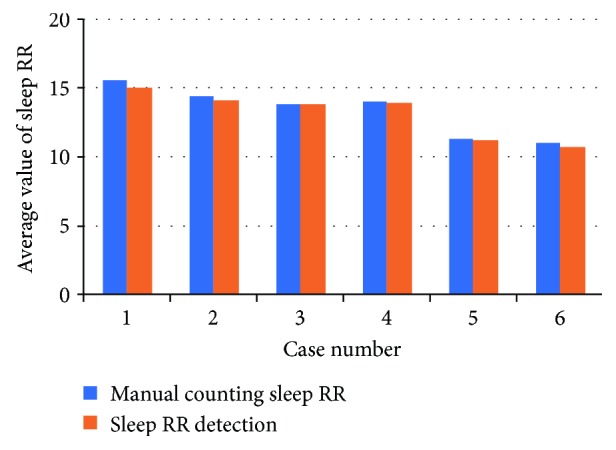
Sleep RR statistic average values of the six cases.

The sleep RR value per minute is computed by counting the number of the segmented breathing cycles. The average values of the sleep RR index of each case are expressed by the bar graph shown in Figure 7. The blue bars in the left show the manual counting results and the red bars in the right show the average sleep RR via the proposed detection method.

It is known that the sleep RR of healthy young men is from 13 to 15 times per minute. And the sleep RR of the OSA case is the slowest among the entire tester which is related to the age and presence of the OSA disease. Specially, the sleep RR of case 5 is closed to that of the OSA case (case 6). Hence, these two cases will be analyzed in detail in the following.

**Figure 8:**
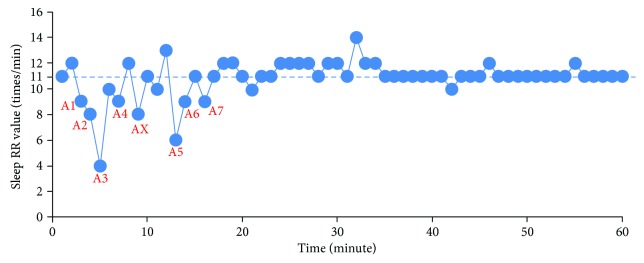
Sleep RR values in the OSA case (number 6).

The plot of the sleep RR value of the OSA case (number 6) in one hour is shown in Figure 8. In order to detect the apnea events, a threshold value *T*_RR_ is set by

(7) $$\begin{array}{c} T_{RR}=RR_{stable}-10*\frac{{RR}_{stable}}{60}, \end{array}$$

where RR_stable_ is the stable or normal respiratory rate in sleeping. The apnea should last more than 10 seconds according to the clinical definition. In another explanation, 10 seconds can be counted as 10∗RR_stable_/60 times/min. Based on the result in Figure 8, the RR_stable_ is 11 times/min; therefore, 10∗RR_stable_/60 is calculated as 1.8 times/min and *T*_RR_ is around 9 times/min. It is found that seven points, denoted by A*i*, are the satisfying condition RR < *T*_RR_ as shown in Figure 8.

**Figure 9:**
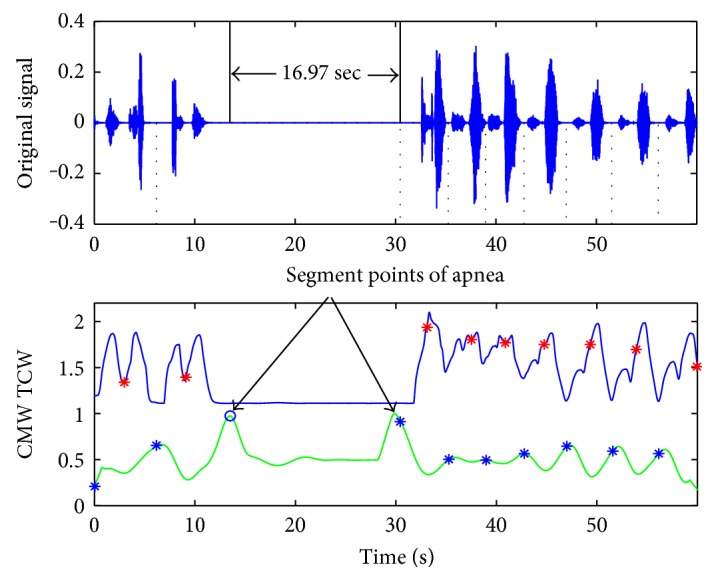
Extraction of time duration for apnea.

**Figure 10:**
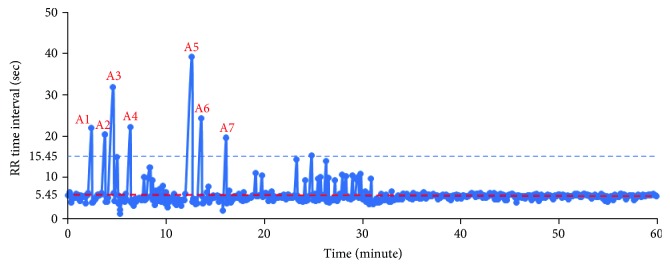
Time duration values of each segmented breath cycle for the OSA case.

In another way to describe the apnea event detection, we can calculate the RR time interval dd(*i*) of the segmentation directly. As the results shown in Figure 6, since each segmented part contains a breathing signal, the apnea pause time can be calculated as dd(*i*) − 60/RR_stable_ as shown in Figure 9.  Figure 10 shows the time duration values dd(*i*) of each segmented breathing cycle. It shows that the stable or normal breathing cycle is about 5 seconds and the longest apnea is about 40.

**Figure 11:**
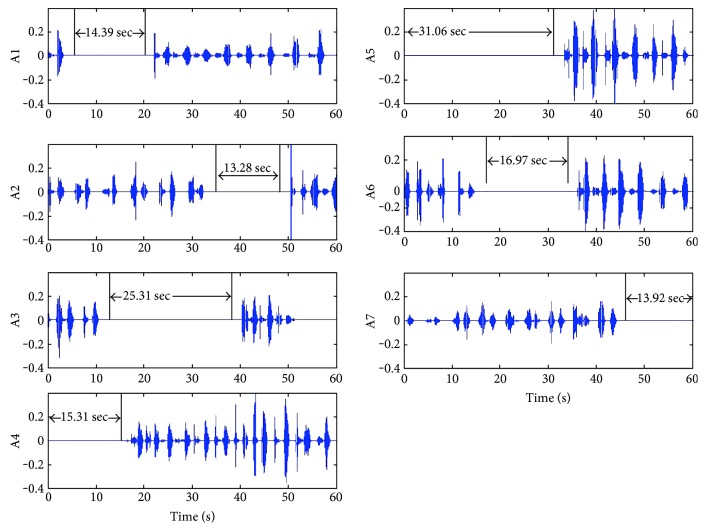
Sleep breathing sound signal waveforms with apnea events (number 6).

Figure 11 shows the pause time calculation results at apnea event points A1 to A7 of Figure 8. The pause time durations of A1 to A7 are 14.39 s, 13.28 s, 25.31 s, 15.31 s, 31.06 s, 16.97 s, and 13.92 s, respectively. Therefore, there are 7 apnea events lasting more than 10 s; the tester might be identified as having mild OSA because of AHI = 7. The sleep RR detection will be acquired for more times of all-night monitoring in order to get more accurate results.

**Figure 12:**
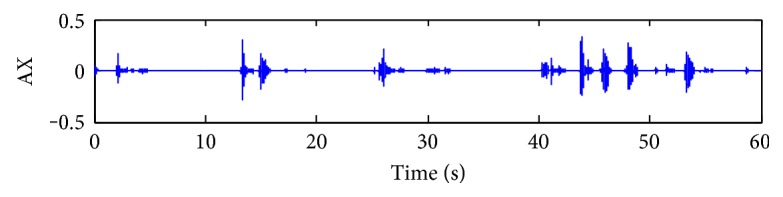
A case of abnormal sleep breathing sound signal waveforms (hypopnea case).

In addition, the signal waveform of the AX section is shown in Figure 12. Since the largest breathing pause is about 7 s, the AX section can be diagnosed as the hypopnea case, a kind of abnormal sleep breath. The abnormal breathing cycles will be meaningful for sleep monitoring.

**Figure 13:**
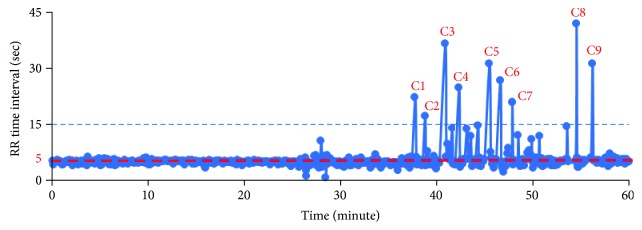
Time duration values of each segmented breath cycle for case number 5.

As mentioned in Figure 7, the data of case number 5 is from a young student and its statistic average value of sleep RR is closed to that of OSA. In the same way, the plot of the time duration values dd(*i*) of each segmentation is shown in Figure 13. The stable breathing cycle lasts about 5 seconds, and nine breathing cycles with apnea are detected.

**Figure 14:**
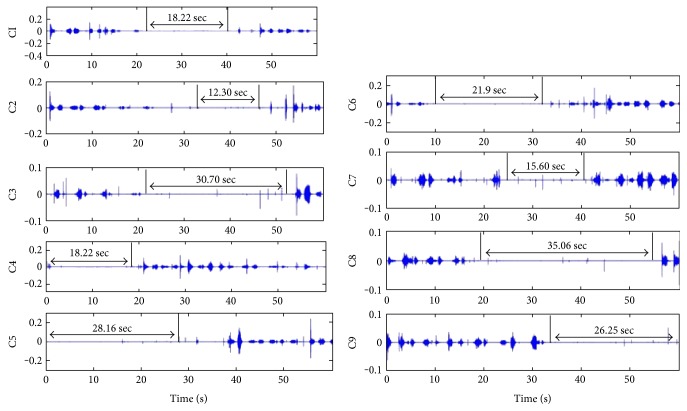
Sleep breathing sound signal waveforms with apnea events (number 5).

The breathing sound signal waveforms with apnea are displayed in Figure 14. Although there are lots of noise chips during the apnea duration and the intensity of the breathing changes greatly, the breathing cycle can be segmented correctly and the apnea can be extracted successfully. It shows that the proposed method has high anti-interference and accuracy on signal segmentation and apnea event extraction.

## 6. Conclusion

Sleep RR is one of the significant human vital signs. The sleep RR and intensity are changed a lot during the whole-night monitoring, and the real-time RR detection will be influenced by strong volume noise. This paper utilizes the characteristic moment waveform for sleep RR detection from the sleep breathing sound signal which is acquired by a wearable sound device. At the first part, the enhanced preprocessing method is applied to reduce the amplitude contrast of the original recording signal. The accuracy of the sleep RR detection and the adaptation of the time scale parameters for different individuals have been improved. According to the results of the experiment, the successful rate of the sleep RR detection can reach to 98.40%. And the sleep breathing sound of subjects with OSA disease can be analyzed easily by the sleep RR value. Moreover, the time interval of apnea can be calculated by the breathing cycle segmentation based on the characteristic moment waveform. The proposed sleep RR detection method is effective for the sleep condition monitoring and OSA disease analysis.

## Figures and Tables

**Table 1:** Experimental data.

Case number	1	2	3	4	5	6	Total
Test time (min)	57	62	85	50	60	60	374
Test cycle number (manual counting)	890	891	1177	702	678	663	5001

**Table 2:** Detection results of the respiratory cycle segmentation.

Case number	Without preprocessing		With preprocessing	
	Cycle number	Successful rate (%)	Cycle number	Successful rate (%)
1	849	95.39	872	97.98
2	851	95.51	865	97.08
3	1156	98.22	1172	99.58
4	683	97.29	694	98.86
5	667	98.38	672	99.12
6	617	93.06	646	97.44
Total	4823	96.44	4921	98.40

Successful rate = segmented cycle number/test cycle number by counting manually.
